# Association of parental adverse childhood experiences with offspring sleep problems: the role of psychological distress and harsh discipline

**DOI:** 10.1186/s13034-024-00796-y

**Published:** 2024-09-09

**Authors:** Yantong Zhu, Gengli Zhang, Shuwei Zhan

**Affiliations:** 1 Faculty of Educational Science, Anhui Normal University, Wuhu, China; 2 School of Education, Central China Normal University, Wuhan, China; 3 Faculty of Educational Science, Anhui Normal University, 2 Beijng Dong Lu, Wuhu, Anhui China

**Keywords:** Parental adverse childhood experience, Psychological distress, Harsh discipline, Sleep problems

## Abstract

**Background:**

Sleep problems are common in early childhood and may be affected by parental adverse childhood experiences (ACEs). However, few studies have examined the longitudinal effect of parental ACEs on offspring sleep problems and the underlying mechanism. This study examined parents’ psychological distress and harsh discipline (psychological aggression and corporal punishment) as mediators in the longitudinal pathway from parental ACEs to offspring sleep problems.

**Methods:**

The participants included 617 3-year-old children (mean age of 43.13 months, SD = 3.82) and their parents (mean age of 33.24 years, SD = 4.01) from Wuhu, China. The participants completed an online questionnaire on ACEs, psychological distress, and demographic characteristics in September 2022 (Time 1). Parents completed another online questionnaire in September 2023 (Time 2) on harsh discipline and offspring sleep problems. A path model was used to examine the associations.

**Results:**

Parental ACEs did not directly predict offspring sleep problems. Psychological distress (β = 0.041, 95% CI [0.005, 0.111]) and harsh discipline in the form of psychological aggression (β = 0.019, 95% CI [0.006, 0.056]) separately mediated the relationship between parental ACEs and offspring sleep problems. Psychological distress and psychological aggression also played a serial mediating role in the association of parental ACEs with offspring sleep problems (β = 0.014, 95% CI [0.007, 0.038]).

**Conclusions:**

Our findings showed the importance of psychological distress and psychological aggression in the intergenerational effect of trauma on offspring sleep problems. Specific interventions aimed at improving mental health and parenting practices should be provided for parents who were exposed to ACEs.

## Background

Adverse childhood experiences (ACEs) refer to traumatic events that occurred before age 18, such as child maltreatment and household dysfunction [1], and have a detrimental effect on an individual’s lifelong health [2]. Chinese populations have a high prevalence of ACEs, which have been reported to exposure at least one ACE range from 31 to 93.5% [3]. Recently, researchers have investigated the transmission of ACEs across generations and the negative effects of parental ACEs on children’s health [4]. Sleep problems are common in early childhood, and a national population-based cohort study indicated sleep problems are prevalent among Chinese preschoolers and more prevalent than in Western countries [5]. According to a recent meta-analysis, the prevalence of sleep problems among children in mainland China has increased over the last two decades, with 38.9% of preschool children experiencing sleep problems [6]. Children with sleep problems tend to be associated with childhood obesity [7], poor cognitive function [8], and more behavioral problems [9]. A previous study suggested that parental ACEs may be a potential risk factor of sleep problems in preschool children [10]; however, few studies have examined the longitudinal association of parental ACEs with offspring sleep problems and the underlying mechanism.

### Parental ACEs and offspring sleep problems

A positive correlation between paternal ACEs and offspring sleep problems was found in previous research [10–12]. However, some studies did not find a significant association between parental ACEs and offspring sleep problems (e.g., getting to sleep and staying asleep) [13, 14]. Because most studies were cross-sectional or used baseline data in a cohort study, this may fail to infer causality between variables. Thus, a longitudinal study should be utilized to explore the relationship between parental ACEs and offspring sleep problems because it can provide more accurate data given the participants are involved over time.

### Harsh discipline as a mediator

Harsh discipline is usually defined as physical abuse, psychological aggression, or corporal punishment, and in Chinese society, psychological aggression and corporal punishment are more commonly used [15]. Psychological aggression refers to verbal and symbolic acts used by parents (e.g., shouting, threatening, or yelling) to purposefully cause psychological pain or fear in the child [16]. Corporal punishment refers to the use of physical force with the intention of causing a child to experience pain, but not injury, in order to correct or control the child’s behavior (e.g., spanking, slapping). Within the context of traditional Chinese culture (i.e., Confucian principles), parental authority is valued, and harsh parenting, such as psychological aggression, is quite common [17]. As such, Chinese children perceive harsh discipline from their parents as an appropriate manifestation of parental authority and care. Lansford et al. [18] posited that harsh parenting may adversely affect child outcomes when it is perceived as nonnormative; conversely, if it is perceived as culturally normative, it may not have any negative impact. As indicated by the Chinese proverb, “Beating and scolding is the emblem of love,” parental harsh discipline may be accepted because both parents and children perceive such behavior as indicative of parental involvement, concern, and affection [19]. Harsh parenting in these conditions may not cause children to have adjustment problems [20]. In Western countries, children may interpret harsh discipline as an indication of parental rejection [21]. This perception can potentially result in feelings of anger and hostility, as well as oppositional behavior [22, 23]. This culture difference of harsh discipline may be related to differences in child mental health outcomes [24]. Moreover, previous studies that have examined the effects of harsh discipline on child mental health outcomes have measured harsh discipline as a unidimensional construct as opposed to measuring different types of harsh discipline [25–27], and combined data on corporal punishment and psychological aggression [28, 29]; however, corporal punishment and psychological aggression may have different effects on children’s outcomes [30, 31]. Therefore, we independently included corporal punishment and psychological aggression as parallel mediators in a path model to explore their effects on their effects on sleep problems among preschool children.

According to ecobiodevelopmental theory, ACEs are toxic, stressful experiences [32] that can have a negative effect on a person’s development (e.g., learning, self-regulation). This may cause a parent to have few resources to manage parenting difficulties and consequently lead to harsh discipline [33]. For example, Shin and colleagues [34] found that higher levels of ACEs in mothers was associated with showing less empathy, which increased parent-to-child aggression. Kim et al. [35] found maternal ACEs predicted an increase in dissociative symptoms, which in turn led to more harsh discipline and less positive structure. Emotion regulation may play a mediating role in the relationship between parental ACEs and negative parenting [36], wherein ACEs are linked to low emotion regulation and thereby increase the use of negative parenting strategies.

Moreover, harsh discipline has been found to be related to children’s sleep problems [37]. According to evolutionary perspectives, sleep environments are influenced by the larger social environment that can either enhance or undermine a sense of security, including the family environment [38]. According to the opponent-process theory, sleep is a physiological state characterized by a significant reduction in awareness and responsiveness to the external environment. Consequently, sleep behavior is likely to be facilitated by perceptions of safety and environments when the need for vigilance is minimal [39]. Wong et al. [40] posited that harsh discipline convey rejection, and the experience of rejection leads to vigilance or defensive behavior in close relationships and a sense of alienation from others. Repeated exposure to harsh discipline may act as forms of distress that interfere with children’s ability to relax and achieve restful sleep [41]. Furthermore, harsh parenting may result in heightened stress sensitivity and poor physiological regulation [42, 43]. These physiological disruptions may, in turn, negatively impact sleep [44]. Therefore, harsh parenting may be distressing and interfere with the reduction in vigilance and arousal necessary to achieve optimal sleep [25].

### Psychological distress as a mediator

Psychological distress is a negative psychological response to stressful life events that includes feelings such as anxiety, overwhelmingness, frustration, and sadness [45]. A representative population-based health survey demonstrated a link between ACEs and psychological distress among adults [46]. From a developmental perspective, emotion regulation mechanisms are assumed to be driven by a complex interaction between neuroendocrine system maturation and social learning opportunities, both which may be especially sensitive to childhood adversity [47]. Rudenstine and colleagues [48] found exposure to ACEs can harm different aspects of emotion regulation (i.e., nonacceptance of emotional responses, impulse control difficulties) and increase psychological distress. Makriyianis et al. [49] found early adversities increased inflexibility, which in turn increased psychological distress.

Studies indicate parental psychological distress can affect children’s sleep problems. Parental psychopathology, particularly depression, is a well-known risk factor for childhood maladjustment [50]. For example, El-Sheikh et al. [51] found children’s sleep and wakefulness difficulties were correlated with higher levels of maternal depressive symptoms, whereas less time spent in bed and less minutes of sleep were linked to higher levels of paternal depressive symptoms. de Jong et al. [52] found greater variability in children’s sleep duration over a 24-h period was correlated with higher levels of mothers’ depressive symptoms. Ystrom and colleagues [53] revealed that anxiety and depression in the mother influenced the child’s nocturnal awakenings at 18 months. Therefore, psychological distress may play a mediating role in the relationship between parental ACEs and children’ s sleep problems.

### Multiple mediating roles of psychological distress and harsh discipline

El-Sheikh et al. [51] suggested that the link between parental psychological distress and child sleep disorder problems is a “parent-driven” mechanism. This mechanism is not only the direct effect of parental psychological distress but also the indirect effect of a variety of parental behaviors [54]; thus, parenting is an important underlying mechanism in the relationship between parental psychological distress and children’s sleep problems. Based on family stress models, Arditti et al. [55] found that mothers who experienced cumulative disadvantages tended to report a higher level of psychological distress and exhibited more harsh discipline. Based on previous research, psychological distress and harsh discipline may serially mediate the relationship between parental ACEs and offspring sleep problems. A serial mediation model hypothesizes a causal chain linking of the mediators (psychological distress and harsh discipline) with a specified direction flow (parental ACEs → psychological distress → harsh discipline → offspring sleep problems).

### The current study

Although previous studies suggest the intergenerational transmission of parental ACEs, few studies have examined the longitudinal effect of parental ACEs on offspring sleep problems and the underlying mechanism among Chinese preschool children. In this study, we applied a longitudinal path model to answer the following research questions: (1) Do parental ACEs have a direct effect on offspring sleep problems? (2) Do parental ACEs indirectly predict offspring sleep problems via psychological distress? (3) Do parental ACEs indirectly predict offspring sleep problems via psychological aggression and corporal punishment? (4) Do psychological distress, psychological aggression, and corporal punishment play a serial mediating role in the relationship between parental ACEs and offspring sleep problems?

## Methods

## Participants and procedures

The Wuhu Family Study is a longitudinal study examining intergenerational transmission of parental ACEs and preschoolers’ well-being. Kindergartens in China provide care and education for children age 3 to 6 before they enter elementary school. Beginning in September 2022, we recruited the parents of newly enrolled 3-year-old children from 11 kindergartens in Wuhu city, including both rural and urban areas, to participate in our study. The kindergartens were randomly selected and parents of children with a disability were not included in the study. The principals and teachers of the kindergartens were informed of the study’s objectives. Then we sent invitations to the parents of the children asking if they would like to participate in our study. All parents were informed of the study’s aims and methods, as well as their right to withdraw at any time. After parents provided informed consent they were asked to complete an online questionnaire on the WenJuanXing platform. A total of 839 parents participated in 2022 and were followed. Of these, 222 parent–child dyads were excluded from this study because of incomplete data at 1-year follow-up or refusal to participate in follow-up. The difference in the scores of the drained participants (*N* = 222) and the reserved participants (*N* = 617) at baseline year were non-significant for parental ACEs (t (837) = 0.68, *p* = 0.48), psychological distress (t (837) = 1.18, *p* = 0.12). Listwise deletion is the most commonly utilized method for dealing with missing data; when the assumption of MCAR (Missing Completely at Random) is met, it is known to yield unbiased estimates and conservative result [56, 57]. With all measures included, the test supported missing data at a random pattern, χ^2^ (5) = 2.85, *p* = 0.772. Thus, 617 parent–child dyads were included in the analysis. In September 2022 (T1), the participants completed a questionnaire on demographic characteristics, ACEs, and psychological distress. In September 2023 (T2), the participants completed a questionnaire on harsh discipline and offspring sleep problems. According to previous research, the prevalence of sleep problems among Chinese preschool children was 38.9% [6], based on an error margin of 5%, and a confidence level of 95%, the calculated sample size was ≥ 366.

The mean age and standard deviation (SD) of the children and their parents at baseline was 43.13 ± 3.82 months and 33.24 ± 4.01 years, respectively. The gender of the children was equally distributed: 309 (50.1%) were boys and 308 (49.9%) were girls. Mothers comprised 80.1% (*n* = 494) of the sample, whereas fathers comprised 19.1% (*n* = 123). Participants’ annual family income ranged from below ¥50,000 to above ¥300,000. Table 1 presents the descriptive statistics for the participants.

**Table 1:** Descriptive statistics for all variables

Variables	Category	*n* (%) or Mean ± SD
Child’s age at baseline year age (Month)		43.13 ± 3.82
Child’s month age at baseline year age (Year)		33.24 ± 4.01
Child’s sex	Male	309 (50.1)
	Female	308 (49.9)
Parent’s sex	Father	123 (19.9)
	Mother	494 (80.1)
Father’s occupation	Unemployed, nontechnical workers, and farmers	14 (2.3)
	Semi-technical worker and small business owner	125 (20.3)
	Technical worker and semi-professional	186 (30.1)
	Professional, officer, and owners of mid-sized business	168 (27.2)
	High-level professional and administrators	124 (20.1)
Mother’s occupation	Unemployed, nontechnical workers, and farmers	123 (19.9)
	Semi-technical worker and small business owner	68 (11.0)
	Technical worker and semi-professional	182 (29.5)
	Professional, officer, and owners of mid-sized business	196 (31.8)
	High-level professional and administrators	48 (7.8)
Father’s education level	Primary school or below	2 (0.3)
	Middle school or below	51 (8.3)
	High school or vocational secondary school degree	83 (13.5)
	Vocational college degree	157 (25.4)
	Bachelor’s degree	256 (41.5)
	Master’s degree or above	68 (11.0)
Mother’s education level	Primary school or below	3 (0.5)
	Middle school or below	48 (7.8)
	High school or vocational secondary school degree	79 (12.8)
	Vocational college degree	169 (27.4)
	Bachelor’s degree	263 (42.6)
	Master’s degree or above	55 (8.9)
Annual family income	< 50,000 RMB	26 (4.2)
	50,001–10,0000 RMB	93 (15.1)
	100,001–150,000 RMB	158 (25.6)
	150,001–300,000 RMB	232 (37.6)
	> 300,000 RMB	108 (17.5)
Parental adverse childhood experiences		3.05 ± 1.78
Psychological distress (T1)		4.79 ± 4.95
Corporal punishment (T2)		7.06 ± 11.72
Psychological aggression (T2)		13.03 ± 17.26
Offspring sleep problem (T2)		3.75 ± 2.36

### Measures

#### Parental ACEs

The Chinese version of the Adverse Childhood Experiences International Questionnaire (ACE-IQ) was used at T1 to assess participants’ ACEs [58, 59]. Participants were asked to retrospectively report ACEs before the age of 18 years. The ACE-IQ includes seven categories: emotional neglect (2 items), physical neglect (3 items), emotional abuse (2 items), physical abuse (2 items), community violence (2 items), peer bullying (3 items), and household dysfunction (6 items). Owing to the sensitivity of the topic in China, questions regarding sexual abuse were omitted from the questionnaire [60]. For household dysfunction, the response options are “yes,” which was scored 1, or “no,” which was scored zero. The other 14 items are responded to using a 5-point scale ranging from 1 = *never true* to 5 = *very often true*. If the participant responded “rarely true,” “sometimes true,” “often true,” or “very often true” to any of the items, they received a score of 1. Thus, ACE-IQ scores ranged from 0 to 7. The Chinese version of ACE-IQ has demonstrated good validity and reliability in parents of preschool children [61]. Cronbach’s alpha in the current study was 0.74.

#### Psychological distress

The Chinese version of the Kessler Psychological Distress Scale (K10) was used at T1 to assess psychological distress (combined feelings of anxiety and depression), which has been showed good validity and reliability among Chinese sample [62, 63]. Items were responded to using a 5-point Likert scale ranging from 0 = *never felt* to 4 = *feel all the time*. Total K10 scores range from 0 to 40, with higher scores indicating a greater level of psychological distress. In this study, Cronbach’s α was 0.94.

#### Harsh discipline

The Chinese version of the Parent–Child Conflict Tactics Scale (CTSPC) was used at T2 to assess parental use of harsh discipline [16, 29]. The Chinese version of the CTSPC has five subscales: Nonviolent Discipline (4 items, i.e., Put him/her in “time out” or sent to his/her room), Psychological Aggression (5 items, i.e., Shouted, yelled, or screamed at him/her), Corporal Punishment (6 items, i.e., Spanked him/her on the bottom with your bare hand), Severe Physical Assault (3 items, i.e., Hit him/her with a fist or kicked him/her hard), and Very Severe Physical Assault (4 items, i.e., Burned or scalded him/her on purpose). In this study, we only used the Corporal Punishment and Psychological Aggression subscales. Participants reported how frequently they had engaged in specific behaviors toward their children in the previous six months: never (0), once (1), twice (2), 3–5 times (3), 6–10 times (4), 11–20 times (5), and > 20 times (6). We recoded ratings of 3–6 as the midpoint of each category (i.e., 3 = 4 times, 4 = 8 times, 5 = 15 times, and 6 = 25 times) to determine the frequency of corporal punishment and psychological aggression, based on Liu and Wang’s [64] study. The Chinese version of CTSPC has been widely used in China and has shown good internal consistency in previous studies (Chan 2012; Liu and Wang 2018). The Cronbach’s alphas in this study were 0.74 for Psychological Aggression and 0.70 for Corporal Punishment.

#### Sleep problems

The Chinese version of the Child Behavior Checklist for ages 1.5–5 (CBCL/1.5-5) was used at T2 to assess sleep problems [8, 65]. The CBCL/1.5-5 includes seven syndromes, such as sleep problems, emotionally reactive, anxious/depressed, and aggressive behavior. The Sleep Problem subscale is a 7-item measure assessing dyssomnia (e.g., sleeps less than most children during the day and/or night) and parasomnia (e.g., talks or cries out in sleep). The subscale has been used to assess children’s sleep problems in China and other countries [66, 67]. Items are responded to using a 3-point Likert scale ranging from 0 = *not true* to 2 = *very true or often true*. Higher scores indicate higher levels of sleep problems. The Chinese version of the CBCL/1.5-5 has been used in a previous study with preschool children and showed good validity and reliability [8]. Cronbach’s α was 0.69 in this study.

#### Covariates

According to previous research examining the intergenerational transmission of ACEs [59]. Parents’ gender (1 = male, 2 = female), parents’ age (in years), children’s age (in months), child’s gender (1 = male, 2 = female), and family socioeconomic status (SES) were included as covariates. Family SES included five indicators: father’s occupation and education level, mother’s occupation and education level, and annual family income. The average standardized scores of the indicators were used to represent SES [59].

### Data analysis

First, descriptive and correlational analyses were conducted. Second, independent samples t-test was used to examine differences between father and mother groups in terms of all study variables. Third, we created a path model to examine the relationship between parental ACEs and offspring sleep problems via psychological distress and harsh discipline. A total of 1000 bootstrap samples were used to estimate the 95% confidence interval (CI) for the significance of effects. Parents’ age, parents’ gender, child’s age, child’s gender, and family’s SES were included as covariates in the analysis.

All analyses were performed using SPSS 28 and Mplus 8.6. The comparative fit index (CFI), Tucker-Lewis index (TLI), root mean square error of approximation (RMSEA), and standardized root mean square residual (SRMR) were used to assess the model fit [68–70]. Acceptable model fit (CFI and TLI > 0.90; SRMR and RMSEA < 0.10) and good model fit (CFI and TLI > 0.95; SRMR and RMSEA < 0.08) were defined using standard benchmark values [71].

## Results

### Descriptive statistics and correlational analysis

Table 1 provides the means ± standard deviations for the main variables. Parental ACEs and psychological distress at T1 were 3.05 ± 1.78 and 4.79 ± 4.95, respectively. Corporal punishment, psychological aggression, and offspring sleep problems at T2 were 7.06 ± 11.72, 13.03 ± 17.26, and 3.75 ± 2.36, respectively. Table 2 shows the bivariate correlations among the main variables. They were all positively and significantly correlated with each other. Independent samples *t*-test showed there is significant difference between father and mother sample in terms of ACEs (t = 2.58, *p* < 0.01), corporal punishment (t = 1.99, *p* < 0.05), which indicates that mothers tend to have more ACEs, and use corporal punishment more frequently. No difference in terms of psychological distress (t = − 1.42, *p* > 0.05), psychological aggression (t = 1.20, *p* > 0.05), and offspring sleep problems (t = 0.25, *p* > 0.05) were found.

**Table 2:** Bivariate correlations among the main variables

	1	2	3	4
1. Parental adverse childhood experiences (T1)	--			
2. Psychological distress (T1)	0.356**	–		
3. Corporal punishment (T2)	0.140**	0.170**	–	
4. Psychological aggression (T2)	0.169**	0.232**	0.663**	
5. Offspring sleep problems (T2)	0.142**	0.194**	0.215**	0.269**

**P < 0.01

### Path analysis

**Fig. 1:**
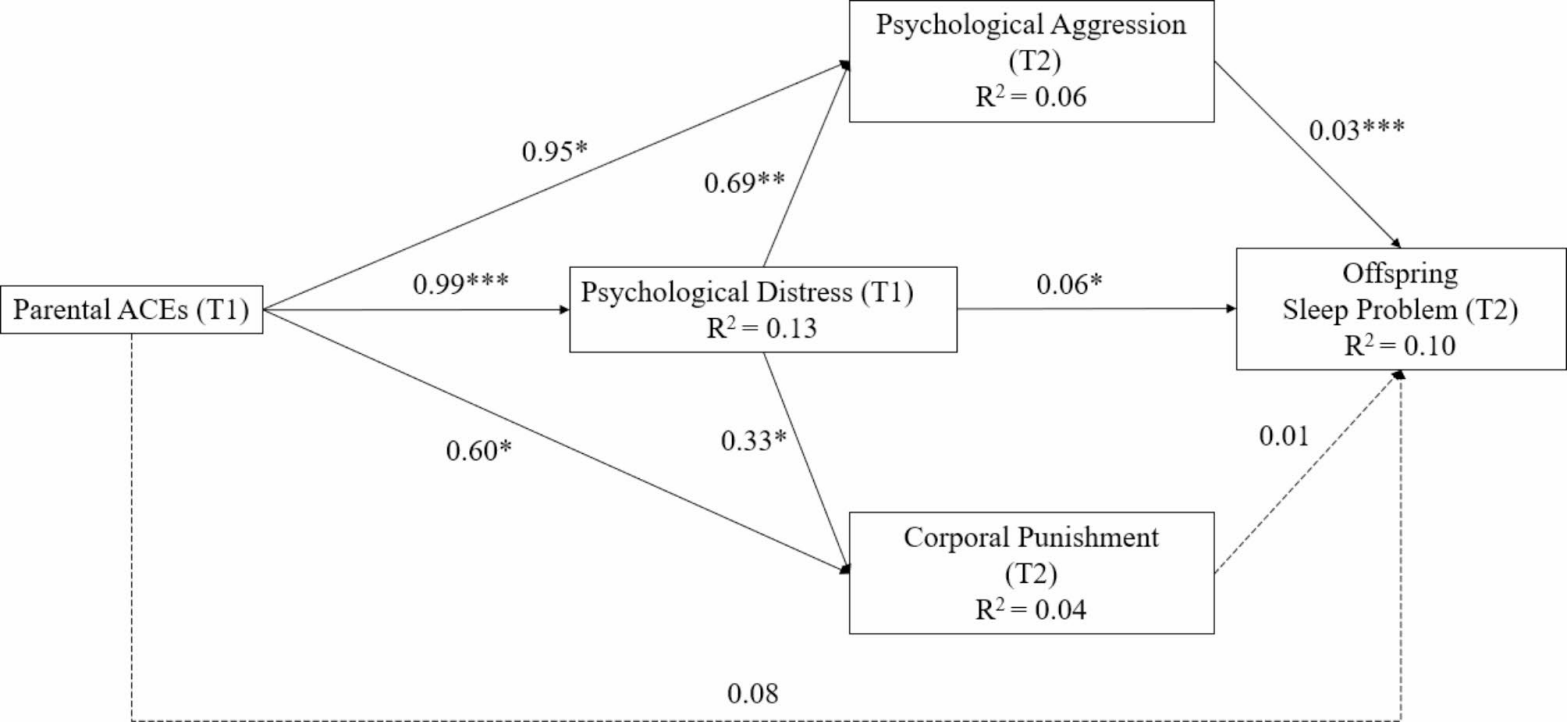
Associations between parental ACEs and offspring sleep problems via psychological distress and harsh discipline. Unstandardized path coefficients are presented next to the arrow. *P < 0.05, **P < 0.01, ***P < 0.001. Psychological distress and corporal punishment were harsh discipline variables. Psychological distress and harsh discipline pertain to adults

Figure 1 shows the relationship between parental ACEs and offspring sleep problems via psychological distress and harsh discipline (corporal punishment and psychological aggression). Results indicated the fit of the path model was acceptable: χ^2^ (15) = 33.64, *p* < 0.01; RMSEA = 0.05, 95% CI [0.02, 0.07]; CFI = 0.97; TLI = 0.93; SRMR = 0.04.

Parental ACEs had no direct effect on offspring sleep problems at T2 (β = 0.063, *p* = 0.173). Parental ACEs significantly and positively predicted psychological distress at T1 (β = 0.356, *p* < 0.001), as well as corporal punishment (β = 0.098, *p* = 0.021) and psychological aggression (β = 0.090, *p* = 0.044) at T2. Psychological aggression was significant related to offspring sleep problems (β = 0.193, *p* < 0.001), whereas the relationship between corporal punishment and offspring sleep problems was not significant (β = 0.063, *p* = 0.284). Parental psychological distress at T1 significantly and positively predicted corporal punishment (β = 0.138, *p* = 0.015), psychological aggression (β = 0.197, *p* < 0.001), and offspring sleep problems at T2 (β = 0.115, *p* = 0.023).

Table 3 shows three significant indirect paths. Parental ACEs had an indirect effect on offspring sleep problems through psychological distress (β = 0.041, 95% CI [0.005, 0.111]) and psychological aggression (β = 0.019, 95% CI [0.006, 0.056]). A significant serial mediation effect was observed from parental ACEs to offspring sleep problems through psychological distress then psychological aggression (β = 0.014, 95% CI [0.007, 0.038]).

**Table 3:** Standardized bootstrap mediation results

Path effect	Standardized β	SE	Bias-corrected 95% CI	
			Lower	Upper
Direct effect				
ACEs → SP	0.063	0.046	− 0.038	0.200
Indirect effects				
ACEs → PD →SP	0.041	0.020	0.005	0.111
ACEs → PA →SP	0.019	0.010	0.006	0.056
ACEs → CP →SP	0.006	0.006	− 0.003	0.030
ACEs → PD →PA → SP	0.014	0.006	0.007	0.038
ACEs → PD →CP → SP	0.003	0.006	− 0.003	0.018

*ACEs* adverse childhood experiences, *SP* sleep problems, *PD* psychological distress, *PA* psychological aggression, *CP* corporal punishment

### Additional analysis

Previous studies have emphasized parenting stress and harsh discipline may vary systematically based on parent gender (Ponnet et al. 2013). For example, compared to fathers, mothers may experience higher levels of stress and used more harsh discipline (Liu and Wang 2015). Therefore, a multiple-group path analysis was conducted to whether there would be a significant difference between paternal and maternal samples in the path model. To examine whether the path coefficients are invariant between the two samples, we compared the unconstrained model (with no constraints across the groups) and the constrained model (where parameters are constrained to be equal across groups). The Chi-square difference test was performed to compare the models.

The result of the Chi-square difference test [Unconstrained model (χ^2^ = 43.02, DF = 33) and constrained model χ^2^ = 34.51, DF = 24)] is not significant (Δχ^2^ = 8.51, DF = 9, *p* = 0.48), meaning the models are invariant across paternal and maternal samples.

## Discussion

This study used a longitudinal path model to examine the intergenerational effect of parental ACEs on offspring sleep problems. This is the first longitudinal study to investigate harsh discipline and psychological distress as mediators in the relationship between parental ACEs and offspring sleep problems. The means of corporal punishment and psychological aggression were similar to previous studies examining the harsh discipline of Chinese preschool parents [72], while sleep problems mean score was lower compared with previous study focusing on Chinese preschool children sleep problems [73]. In the proposed model, parental ACEs did not have a longitudinal direct effect on offspring sleep problems; however, three significant indirect pathways were identified. The results of this study may assist in the development of targeted interventions to prevent the intergenerational cycle of trauma.

First, consistent with Merrill et al.’s [13] study, we found parental ACEs at T1 did not predict offspring sleep problems at T2. This finding suggests that parental ACEs are not likely to be directly related to offspring sleep problems, but possibly indirectly related through parents’ mental health and parenting behaviors. Although a meta-analysis showed that exposure to ACEs increased adult sleep problems [74], Rönnlund et al. [75] found that poor parental sleep was not associated with future sleep problems in children aged 2–6 years. Furthermore, our results showed parental ACEs were significantly related to psychological distress at T1, and corporal punishment and psychological aggression at T2, which is consistent with previous findings [76]. Moreover, psychological distress may harm parental emotional availability [77], emotional availability of mothering at bedtime was significantly associated with regulated childhood sleep patterns [78]. Psychological distress may also contribute to a more inconsistent and permissive parenting style [54], in which parents are unable to effectively monitor and discipline their children’s misbehavior [79], as well as struggle to enforce a consistent bedtime routine and sleep schedule. Inconsistent bedtime routine and sleep schedule may be related to children’s sleep problems [80]. Thus, parents’ exposed to ACEs tend to experience more psychological distress and consequently apply more harsh parenting strategies.

Second, our results showed an indirect pathway between parental ACEs and offspring sleep problems via psychological distress. Early traumatic experiences can cause children’s stress-response system dysregulation, which disrupts neurobiological mechanisms resulting in long term negative effects on mental health [81]. For example, early traumatic experiences can harm HPA axis activity [32]. Individuals who have had recurrent ACEs are more likely to struggle with subsequent stressful events because their stress response system is overburdened making them less able to efficiently manage biological stress processes [80]. Parents with psychological distress may increase the risk of creating a dysfunctional parent–child system [51], in which parents’ emotional dysregulation may disrupt stable sleep patterns in their children [77].

Moreover, we found psychological aggression mediated the relationship between parental ACEs and offspring sleep problems, but not corporal punishment, which is consistent with Miller-Perrin et al.’s [82] study. Chinese parents use psychological aggression more frequently than corporal punishment [30]. Psychological aggression is less violent and therefore may be considered as more suitable and socially acceptable; when it is effective, parents will not use corporal punishment [32]. Thus, parents who have had ACEs may be more likely to affect offspring sleep problems indirectly through psychological aggression than corporal punishment. Miller-Perrin et al. [82] suggested that parental psychological aggression has a central role in predicting psychological adjustment in both children and adults who experience various forms of parental aggression. They examined the relationship between psychological symptoms among young adults and both parental physical and psychological aggression. When combining the different types of parental aggression in one model, psychological aggression was the only type of parental aggression that significantly related to psychological symptoms. Results showed that corporal punishment did not relate to children’s sleep problems, and may be explained by cultural norms for adult corporal punishment [21]. Parental corporal punishment was accepted in Chinese culture because both parents and children perceive such behavior as indicative of parental concern and love [19]. According to Rohner and colleagues [22], in cultures where corporal punishment is perceived as a normative practice, children may not interpret such disciplinary actions as indicators of parental rejection or excessive harshness, and may not cause sleep problems [83].

Lastly, this study revealed psychological distress and psychological aggression serially mediated the association between parental ACEs and offspring sleep problems. Parents exposed to ACEs often experience a higher level of psychological distress, which leads to the use of psychological aggression, and thereby offspring sleep problems. Previous studies have confirmed the intergenerational transmission of harsh discipline through psychological distress [17]. Parents exposure to ACEs may enhance the level of psychological distress, and individuals with psychological distress are more likely to become harsh parents [84]. According to the “parent-driven” mechanism [52], parental psychological distress not only directly influences children’s sleep problems but also indirectly through parenting behaviors.

Our study extends previous research by revealing an underlying mechanism of how parental ACEs affect sleep problems in children. A strength of this study is the use of a longitudinal path model to reveal whether psychological distress and harsh discipline indirectly affect the intergenerational transmission of trauma between parents and their young children. Nevertheless, this study has some limitations. First, although we included fathers in our study, they were a small percentage of the sample. Future research should make efforts to recruit a relatively equal number of fathers and mothers as participants. Second, retrospective reports of ACEs may be subject to recall bias [85]. Third, despite childhood sexual abuse being linked to behavioral problems [86], we did not include it in this study because it is a sensitive topic in China [60]. Doing so could potentially cause adverse reactions in participants due to the shame and sensitivity associated with sexual victimization in China [42]. Fourth, the current study did not measure parental harsh discipline and children’s sleep problem at T1. Controlling for harsh discipline and children’s sleep problems at T1 would help control for residual change and impact the results that can be made pertaining to predictions over time. Furthermore, previous research suggested the bidirectional relationship between parental psychological distress, psychological aggression, and children’s sleep problems [87, 88]. Future research would benefit from controlling for harsh discipline and children’s sleep problems at baseline year and examining the transactional dynamics relationship between parental psychological distress, psychological aggression, and children’s sleep problems. Fifth, all information was reported by one caregiver. We suggest future studies could use different methods, such as behavioral observation to measure harsh parenting, and collect data from both the father and mother of the children. Finally, Our research applied the parent-report CBCL sleep scale to measure children’s sleep problems, we suggest future research use more reliable methods to test children’s sleep (i.e., sleep diary, polysomnography). Moreover, previous research showed that ACEs may affect sleep duration [89], future research should include offspring’s sleep duration when examining the effect of parental ACEs.

## Conclusion

Our study provides important empirical evidence for the underlying mechanism in the relationship between parental ACEs and offspring sleep problems among Chinese preschool children, contributing to an understanding of the intergenerational transmission of trauma. The results revealed that although parental ACEs did not affect offspring sleep problems directly, parental psychological distress and psychological aggression were mediators in this relationship, both separately and sequentially. The results suggest a need to pay attention to parents who have experienced ACEs and highlight the importance of improving levels of mental health and parenting strategies as a means to mitigate the effects of parental ACEs on offspring sleep problems. In response to this finding, implementing targeted intervention programs to support parents with the greatest risk of exposure to ACEs and incorporate trauma-informed programs to promote their mental health and parenting strategies.

## Data Availability

The datasets used and analyzed during the current study are available from the corresponding author on reasonable request.

## Abbreviations

ACEs: Adverse childhood experiences
ACEs: Adverse childhood experiences
ACE-IQ: Adverse childhood experiences international questionnaire
CBCL: Child behavior checklist
CFI: Comparative fit index
CI: Confidence interval
CTSPC: Chinese version of the parent–child conflict tactics scale
K10: Kessler psychological distress scale
SES: Socioeconomic status
TLI: Tucker–Lewis index
RMSEA: Root mean square error of approximation
SRMR: Standardized root mean square residual
